# Mechanisms of Adsorption of Phenoxyalkanoic Herbicides on Fulvic and Humic Acids

**DOI:** 10.3390/ijms252312699

**Published:** 2024-11-26

**Authors:** Tadeusz Paszko, Joanna Matysiak, Claudio A. Spadotto, Patrycja Boguta, Kamil Skic

**Affiliations:** 1 Department of Chemistry, University of Life Sciences, Akademicka 13, 20-950 Lublin, Poland; joanna.matysiak@up.lublin.pl; 2 Embrapa Digital Agriculture, Av. André Tosello, 209, Campinas 13083-886, São Paulo, Brazil; claudio.spadotto@embrapa.br; 3 Institute of Agrophysics, Polish Academy of Sciences, Doświadczalna 4, 20-290 Lublin, Poland; p.boguta@ipan.lublin.pl (P.B.); k.skic@ipan.lublin.pl (K.S.)

**Keywords:** acidic herbicides, adsorption mechanisms, thermodynamics, humic substances, FTIR, molecular modeling

## Abstract

Our recent study demonstrated that fulvic and humic acids are the major contributors to the adsorption of phenoxyalkanoic acid herbicides in soils. At very low pH, the neutral forms of these herbicides are bound directly to fulvic and humic acids, whereas at higher pH, their anionic forms are adsorbed mainly via bridges created by Al^3+^ species. The number of active sorption sites associated with Al^3+^ species complexed with fulvic acids is pH-dependent, whereas the number of corresponding sites in humic acids is pH-independent. Based on the results of the FTIR analysis, research into adsorption thermodynamics, and molecular modeling, an attempt was made in the present study to explain the adsorption mechanisms of six phenoxyalkanoic herbicides used currently in the European Union on the surfaces of the above fractions of humic substances. The obtained values of standard enthalpy (${\Delta H}^{0}$) for the adsorption of the anionic forms of phenoxyalkanoic herbicides on fulvic or humic acids complexed with Al^3+^ were in the range of physical adsorption, i.e., from −8.4 kJ/mol to −2.9 kJ/mol for the former, and from −5.3 kJ/mol to −2.4 kJ/mol for the latter. The study demonstrated that the neutral forms of phenoxyalkanoic herbicides were bound to humic substances mainly via H-bonds, π-π stacking interactions, and hydrophobic interactions. Al^3+^ species were complexed with fulvic and humic acids to form outer-sphere complexes. Ternary outer-sphere complexes were also created between the anionic forms of phenoxyalkanoic acid herbicides and positively charged Al^3+^ species complexed with fulvic acids. The mechanisms of adsorption on humic acids involved a ligand exchange between a loosely bound hydroxyl group of hydrolyzed Al^3+^ complexed with this adsorbent and the anionic form of the herbicide. However, in this case, adsorption took place only in the presence of sufficiently strong hydrophobic and π-π stacking interactions supported by H-bonds. These findings elucidate why phenoxyalkanoic herbicides are mobile in the soil profile and are often rapidly degraded in soils.

## 1. Introduction

Phenoxyalkanoic acid herbicides (PAAHs; Table 1) were first used in the 1940s [1] and since then, they have been widely applied throughout the world [2]. Although PAAHs are post-emergence herbicides, significant amounts of these compounds reach the soil surface, surface water, and groundwater, and the processes of their adsorption and degradation should be analyzed to predict pesticide behavior and environmental impact. A pan-European study on the occurrence of polar organic persistent pollutants in groundwater demonstrated that the frequency of PAAH detection ranged from 3.7% to 13.4%. MCPP and DCPP were found in the highest concentrations; in some samples, their concentrations exceeded 0.1 μg/L, i.e., the groundwater quality standard for individual pesticides [3]. In turn, MCPP and 2,4-D were detected very frequently (~52% and 42%, respectively) in European river water samples [4]. A recent study by Wu et al. [2] and our previous study [5] suggest that the transport of PAAHs adsorbed on the surface of water-soluble humic substances contributes significantly to the contamination of groundwater and surface water with these herbicides.

In the top soil layer, PAAHs are adsorbed mainly on organic matter, and sorption is negatively correlated with soil pH [6,7]. The contribution of soil inorganic components to the overall sorption of PAAHs increases with soil depth. The adsorption of PAAHs in soils decreases in the following order: 2,4-DB > MCPB >> 2,4-D > MCPA >DCPP-P > MCPP-P [5,8]. Taking into account the data from European Union dossiers [9], the adsorption of 2,4-DB expressed by the adsorption distribution coefficient normalized to organic carbon (*K_OC_*) can exceed 1000 mL/g, whereas the maximum adsorption of MCPP-P is around 150 mL/g. PAAHs are quickly adsorbed in soils, and in batch experiments, adsorption usually reaches equilibrium within 4 to 12 h [8]. PAAHs are also easily desorbed from soils. In soils with an organic matter content of >1%, typically 40% to 80% of the adsorbed PAAHs can undergo desorption. In soils with an organic matter content of <0.3%, the effectiveness of desorption reached 100% [8]. Thermodynamic parameters have been rarely determined for the adsorption of PAAHs in soils. In the work by Shariff [10], the standard enthalpy (${\Delta H}^{0}$) of the adsorption of 2,4-D in soils ranged from −28.6 kJ/mol to −18.5 kJ/mol. These values indicate that physical adsorption, including hydrophobic sorption, van der Waals interactions, H-bonding, water-bridging, and/or anion exchange, should be the predominant process for 2,4-D [8].

Humic substances play an important role in protecting the environment against contamination because the contaminants are incorporated into the matrix of humic substances through different mechanisms [11]. The chemical behavior of humic substances is determined mostly by carboxylic and phenolic functional groups, and humic molecules have a negative charge that is pH-dependent due to the partial dissociation of these acid groups [12]. Fulvic acids (FAs) and humic acids (HAs) are humic substances with different solubility in water. Fulvic acids are water-soluble at any pH, whereas HAs are water-soluble at alkaline pH. Therefore, 0.1 M NaOH or 0.1 M Na_4_P_2_O_7_ solutions are most often used to extract these fractions from soils, and the obtained extracts are acidified to pH 1.0–1.5 with HCl, which leads to the sedimentation of HAs [13,14]. The role played by FAs and HAs in the sorption of PAAHs in soil has not been fully elucidated to date due to the limited number of studies investigating the sorption of PAAHs on isolated fractions of humic substances [12,15,16,17,18,19].

According to Haberhauer et al. [20], Kah and Brown [21], Ćwieląg-Piasecka et al. [19], as well as our recent study [5], the most important interactions between the neutral forms of PAAHs and humic substances should involve H-bonding as well as van der Waals and hydrophobic interactions. In a study by Khan [15], the ${\Delta H}^{0}$ of 2,4-D adsorption on HAs at pH 3.3–3.6 (at this pH, the adsorption of its neutral form should be dominant) was 5.69 kJ/mol, i.e., remained in the physical adsorption range [22,23]. For the anionic forms of PAAHs, electrostatic interactions should also occur with their deprotonated carboxyl groups. Based on the pKa (negative logarithm of the dissociation constant; Table 1) values of the six PAAHs that have been authorized for use in the European Union [24], as well as the assumption that most soils have a pH of 5 to 8, only the anionic forms of 2,4-D, MCPA, DCPP-P and MCPP-P, and the anionic and neutral forms of 2,4-DB and MCPB are adsorbed at pH > 5. Our recent study [5] has revealed that at low pH, FAs and HAs adsorb the neutral forms of all PAAHs, whereas at pH > 5, pure FAs are unable to adsorb the anionic forms of any PAAHs, and pure HAs can adsorb only the anions of 2,4-DB and MCPB. For example, the *K_OC_* values for MCPB, 2,4-D, and MCPP-P adsorbed on FAs examined in the current study reached 2482.2, 937.0, and 814.0 mL/g, respectively, at pH 2.9 and 548.8, 45.8, and 3.5 mL/g, respectively, at pH 5.7. In turn, the *K_OC_* values for MCPB, 2,4-D, and MCPP-P adsorbed on HAs examined in this study reached 659.1, 108.2, and 101.6 mL/g, respectively, at pH 2.9, and 170.7, 0.0, and 0.0 mL/g, respectively, at pH 7.1.

**Table 1:** Structure and selected physicochemical properties of the examined PAAHs.

Name		2,4-DB		DCPP-P		2,4-D		MCPB		MCPP-P		MCPA	
CAS name	Structural formula	4-(2,4-dichlorophenoxy)butanoic acid	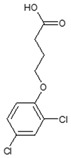	(2R)-2-(2,4-dichlorophenoxy)propanoic acid	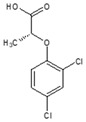	2-(2,4-dichlorophenoxy)acetic acid	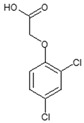	4-(4-chloro-2-methylphenoxy)butanoic acid	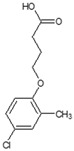	(2R)-2-(4-chloro-2-methylphenoxy)propanoic acid	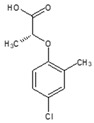	2-(4-chloro-2-methylphenoxy)acetic acid	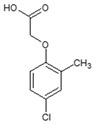
Mol wt.		249.1		235.1		221.0		228.7		214.6		200.6	
*pK_a_* (SD) ^1^		4.74 (0.05)		2.98 (0.02)		2.93 (0.01)		5.15 (0.05)		3.14 (0.01)		3.06 (0.01)	
C log P ^2^		3.42		3.04		2.73		3.39		3.01		2.70	

^1^ Determined spectrophotometrically (20 °C, I = 0.01) according to Albert and Serjeant [25]. ^2^ Calculated hydrophobicity partition coefficient [26,27].

However, herbicide adsorption in soil depends on the interactions between organic matter, Fe and Al oxyhydroxides, and clay minerals [16,20,28]. According to Piccolo and Stevenson [29], HAs and FAs can interact with metal ions to form metal–organic complexes in soils. Studies on the adsorption of 2,4-D [30] and DCPP [18] on FAs after coagulation with Al^3+^ indicated that the anionic forms of these herbicides were bound to FAs mostly via an Al^3+^ bridge. Larrivee et al. [30] provided evidence that metal ions (Al^3+^ and Pb^2+^) interact strongly with organic complexing agents (FAs and 2,4-D) and suggested that negatively charged FA components can interact with negatively charged herbicides (such as 2,4-D) through positively charged metal ion bridges. According to Elkins et al. [18], Al^3+^ species can form complexes with DCPP and/or FAs, and complexation reactions play an important role in decreasing herbicide degradation and mobility in soils and in determining the environmental fate and bioavailability of aluminum. Jin et al. [31] investigated the interactions between HAs and an Al^3+^ coagulant and demonstrated that at pH 5, the activated functional groups of HAs formed complexes with Al^3+^ species. However, at pH 7, surface complexation occurred, and the activated functional groups were absorbed on amorphous aluminum hydroxide (Al(OH)_3_).

In our recent study [5], when FAs were coagulated with sufficiently large amounts of Al^3+^, the adsorption of the anionic forms of all PAAHs increased and reached a maximum at pH~5.0–5.5, and it decreased to zero at pH~7.0–7.5. The adsorption of large amounts of Al^3+^ species by HAs resulted in high adsorption of 2,4-D, MCPA, DCPP-P, and MCPP-P anions, whereas the adsorption of 2,4-DB and MCPB anions was much lower in comparison with their adsorption on pure HAs. Moreover, the adsorption of the anionic forms of PAAHs on HAs was similar in the pH range of ~5 to 7.5. For example, the *K_OC_* values for MCPB, 2,4-D, and MCPP-P adsorbed at pH 5.7 in the examined in this study FAs complexed with Al^3+^ species reached 7923.1, 825.1, and 1123.2 mL/g, respectively. In turn, the *K_OC_* values for MCPB, 2,4-D, and MCPP-P adsorbed at pH 7.1 on the examined HAs complexed with Al^3+^ species reached 59.4, 1168.4, and 1139.5 mL/g, respectively.

Our previous study involved mainly batch experiments which were not sufficient to explain the observed adsorption phenomena. These mechanisms should be further explored because they play a key role in the retention of PAAHs in soil and their bioavailability to soil bacteria. Therefore, the aim of the present study was to elucidate the adsorption mechanisms between FAs or HAs, Al^3+^ species, and the molecular and anionic forms of PAAHs based on the results of Fourier-transform infrared (FTIR) spectroscopy, the values of the thermodynamic parameters of PAAH adsorption, and molecular modeling.

## 2. Results

### 2.1. FTIR Results

#### 2.1.1. FTIR Spectra of Pure FAs and HAs, and After the Adsorption of Al^3+^ Species

The FA fraction examined in this study was characterized by a high content of carboxyl groups, aliphatic and aromatic hydrocarbons, and a moderate content of lignin (Table 2). In the HA fraction, the content of carboxyl groups and aliphatic hydrocarbons was much lower, whereas the content of aromatic hydrocarbons was much higher.

**Figure 1:**
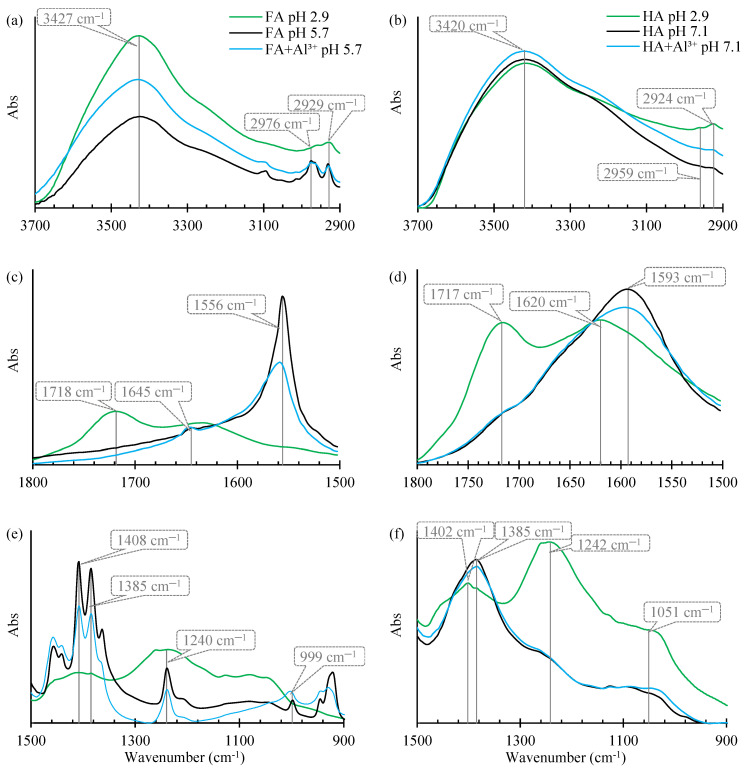
FTIR spectra of FAs (pH 2.9 and 5.7), FAs+Al^3+^ (pH 5.7), HAs (pH 2.9 and 7.1), and HAs+Al^3+^ (pH 7.1) at (**a**,**b**) 3700–2900 cm^−1^, (**c**,**d**) 1800–1500 cm^−1^, and (**e**,**f**) 1500–900 cm^−1^.

The FTIR spectra of FAs and HAs at pH 2.9 were characterized by a broad band (3418–3427 cm^−1^; Figure 1 and Table 3) attributed mainly to H-bonded –OH, a visible band of –CH_2_– (2924–2929 cm^−1^), bands of the –COOH group (1717–1718 cm^−1^ and 1236–1242 cm^−1^), and a broad band associated with a strongly H-bonded C=O group (1620–1633 cm^−1^). When pH was adjusted to 5.7 (FAs) or 7.1 (HAs), the –COOH group bands at 1717–1718 cm^−1^ disappeared and were significantly reduced to 1236–1242 cm^−1^ (the band at ~1240 cm^−1^ was formed by the C-O stretch of aryl ethers [33,34]). Moreover, the bands were detected at 1556 cm^−1^, and 1385 cm^−1^ for FAs, and at 1593 cm^−1^ and 1385 cm^−1^ for HAs due to the transformation of the –COOH group into its anionic form. The band at 1402–1410 cm^−1^ was related to O–H and C–O stretching of phenols [34,35].

The addition of AlCl_3_ led to the coagulation of FAs. In FA+Al^3+^ samples at pH 5.7, a significant increase in peak height was observed at 3431 cm^−1^ and 1003 cm^−1^ (Figure 1). In HA+Al^3+^ samples at pH 7.1, peak height increased at 3423 cm^−1^ and 1049 cm^−1^, and the shoulder increased between 1080 cm^−1^ and 940 cm^−1^. Moreover, in FA+Al^3+^ samples, peak height was reduced at 1558, 1408, and 1385 cm^−1^. The peak at 1558 cm^−1^ was slightly shifted toward higher frequencies (Table 2), and the peak at 1238 cm^−1^ was shifted toward lower frequencies.

Jin et al. [31] reported highly similar results in an experiment investigating the coagulation of HAs by Al^3+^. At pH 5, peak absorbance decreased at 1717 cm^−1^ (in the present study, absorbance decreasing was visible in FA samples), the peak disappeared at 1247 cm^−1^ (in the present study, a very small but visible decrease was noted in FA samples), and the bands of asymmetric and symmetric –COO^−^ stretches were shifted toward higher frequencies. The observations made by Jin et al. [31], Elkins et al. [18], and Simonsson [36] and the results of this study indicate that Al^3+^ species are adsorbed by binding with dissociated carboxylic groups and by H-bonds with the –OH groups of alcohols and phenols. In the current study, the –OH groups of polysaccharides (bands at 1005 and 1048 cm^−1^) also contributed to the adsorption of Al^3+^ species by FAs and HAs. In the work of Boguta et al. [37], band absorbance increased at 1033–1039 cm^−1^ after the adsorption of Fe^2+^ species on HAs. 

#### 2.1.2. FTIR Spectra of FAs and FAs+Al^3+^ After the Adsorption of PAAHs

**Figure 2:**
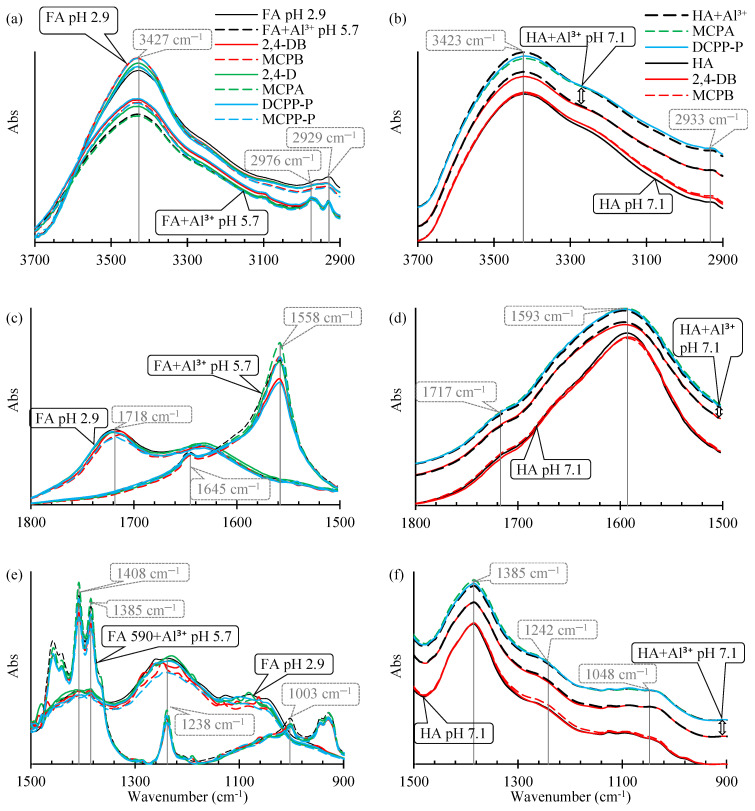
FTIR spectra at 3700–2900 cm^−1^ (**a**,**b**), at 1800–1500 cm^−1^ (**c**,**d**), and at 1500–900 cm^−1^ (**e**,**f**) of pure FAs (pH 2.9), FAs+Al^3+^ (pH 5.7), HAs and HAs+Al^3+^ (pH 7.1), and the spectra after the adsorption of selected PAAHs. The spectra of HAs+Al^3+^, HAs+Al^3+^+MCPA, and HAs+Al^3+^+DCPP-P were shifted up to avoid overlap.

The adsorption of PAAHs on pure FAs at native pH 2.9 decreased slightly the bands at around 1718 cm^−1^ (Figure 2c). In MCPB and 2,4-DB spectra, characterized by the highest adsorption, a minor shift to 1714 cm^−1^ was observed. The adsorption of MCPB, 2,4-DB, and MCPP-P also reduced the band at 1236 cm^−1^. In the infrared spectra, little or no band shift is expected for outer-sphere surface complexes, and band shifts to higher wavenumbers are expected for inner-sphere complexes [31]. These observations point to the presence of intermolecular H-bonds of –COOH groups and other carbonyl groups of FAs with an –OH group of the undissociated carboxylic groups of PAAHs.

**Table 3:** Band assignments of FTIR spectra for the examined PAAH adsorbents.

Location (cm^−1^)						Assignment (Comments)	References
FAs pH 2.9	FAs pH 5.7	FAs+Al^3+^ pH 5.7	HAs pH 2.9	HAs pH 7.1	HAs+Al^3+^ pH 7.1		
3427	3425	3431	3418	3420	3423	O–H stretching of hydroxyl groups involved in H-bonds, N–H stretching of amines (weaker)	[38,39]
2958	2976	2976	2959	Nd ^1^	Nd	Asymmetric –CH_3_ stretching	[38]
2929	2932	2930	2924	Nd	2933	Asymmetric –CH_2_– stretching	[38]
1718	Nd	Nd	1717	Nd	Nd	C=O stretching in –COOH, ketones and esters	[29,38]
1633	1645	1645	1620	Nd	Nd	strongly H-bonded C=O, aromatic –C=C– stretching,	[29,31,38]
Nd	1556	1558	Nd	1593	1593	Asymmetric stretch of –COO^−^, aromatic –C=C– stretching	[29,31,38]
Nd	1408	1408	1402	Nd	Nd	O–H and C–OH stretching of phenols, aromatic –C=C– stretching	[34,35]
1385	1385	1385	1385	1385	1385	Symmetric –COO^−^ stretching,	[29,31,38]
1236	1240	1238	1242	Nd	Nd	C–O stretching and O–H deformation in –COOH, C–O stretching of ethers and alcohols/phenols	[29,31,33,34,38]
Nd	999	1003	1051	Nd	1049	C–OH stretching in polysaccharides	[35,37,38]

^1^ Nd—not determined.

The adsorption of PAAHs on FAs at a native pH of 2.9 led also to an increase in the absorbance of the band of –OH groups at 3427 cm^−1^ (Figure 2). In comparison with the band for pure FAs, the greatest increase was observed for MCPB and 2,4-DB. The above can be attributed, among other things, to the fact that at pH 2.9 MCPB and 2,4-DB occurred almost exclusively (≥99%) in neutral forms, whereas the neutral forms of the remaining PAAHs were noted in the range of 57–69%. The spectra of FA samples with adsorbed PAAHs were decreased in the range of 3300–3000 cm^−1^ and 3000–2900 cm^−1^ (Figure 2a). The higher part of this shoulder (around 3270 cm^−1^) is associated with phenolic and/or alcoholic –OH groups, and the lower part (2976 and 2930 cm^−1^) is associated with aliphatic –CH_3_ and –CH_2_– groups [29]. Moreover, the spectra associated with C–OH stretching in alcohols in the range of 1180–1040 cm^−1^, and the spectra associated mainly with C–OH stretching in polysaccharides in the range of 1040–1000 cm^−1^ were decreased [35,37,38]. The above points to the presence of H-bonds and hydrophobic and/or van der Waals interactions between FAs and PAAHs.

Wu et al. [2] found a similar decrease in peak absorbance at ~3400 cm^−1^ and similar changes in peaks at ~3000 cm^−1^ and ~1730 cm^−1^ for MCPA adsorption on the hydrophobic acid fraction of dissolved organic matter, which can be largely identified with FAs.

After the adsorption of PAAHs on FAs+Al^3+^ at pH 5.7, noticeable changes were observed in the bands identified at 1558, 1408, and 1385 cm^−1^, and they were attributed to vibrations in –COO^−^, as well as O–H and C–OH in phenols. The changes noted in the band at 1238 cm^−1^ were associated with C–O stretching in aryl ethers and alcohols/phenols (Table 3). The above bands had similar patterns: The absorbance for MCPA was strongest, and the absorbances for DCPP-P, 2,4-DB were weakest. In our previous study [5], the sorption sites associated with the Al^3+^ species adsorbed by FAs had the weakest affinity for MCPA and 2,4-D, and the strongest affinity for 2,4-DB, MCPB, and DCPP-P. Therefore, the weaker the absorbance of the bands for –COO^−^ with the adsorbed Al^3+^ species, the stronger the interaction with PAAHs.

The absorbance of FA+Al^3+^ coagulate samples at 3431 cm^−1^ was enhanced after the adsorption of PAAHs at pH 5.7, and the greatest increase was observed for DCPP-P, 2,4-DB, MCPB, MCPP-P, and 2,4-D. For MCPA, no visible differences were observed compared to the pure coagulate. This observation suggests that the number of H-bonds increased after the addition of five PAAHs. Moreover, the spectra in the range of 1220–1100 cm^−1^ (C–OH stretching in alcohols and phenols) were decreased for all PAAHs. In addition, the band of –OH groups in polysaccharides, which increased after the addition of Al^3+^ to FAs (999 cm^−1^ in Figure 1e), decreased after the addition of all PAAHs (1003 cm^−1^ in Figure 2e). The above findings indicate that –OH groups in alcohols, phenols, and polysaccharides are involved in the adsorption of PAAHs.

Al^3+^ species are adsorbed by FAs mainly via a cation-exchange mechanism, which, in pure FAs, involves the release of H^+^ from the –COOH group [36,40]. Simonsson [36] reported that some of the Al^3+^ was adsorbed by FAs in a partially hydrolyzed form. This observation also indicates that some surface sites associated with the adsorbed Al^3+^ can be positively charged, depending on the pH and the amount of adsorbed Al^3+^. At pH 5.7, Al(OH)_2_^+^ is the dominant form in aquatic solutions, and only trace amounts of Al^3+^ are present [41]. At this pH, 78.0% of MCPB, 90.1% of 2,4-DB, and ≥99.7% of other PAAHs are present in anionic form. These results suggest that Al^3+^ bridges and H-bonds are the main mechanisms by which the anionic forms of PAAHs are adsorbed on FAs.

#### 2.1.3. FTIR Spectra of HAs and HAs+Al^3+^ After the Adsorption of PAAHs

**Figure 3:**
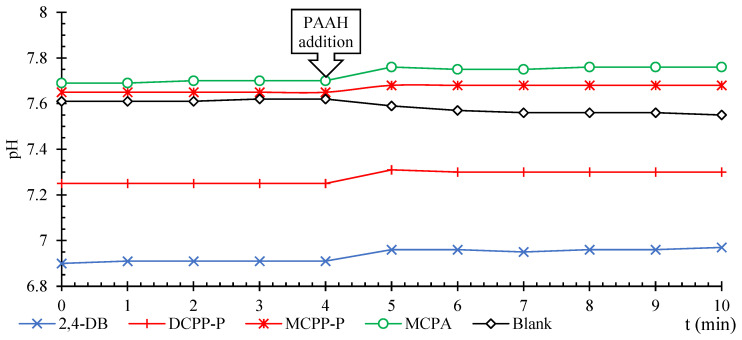
Changes in pH after PAAHs or the blank solution were added to the suspensions of HAs complexed with Al^3+^ species. The added solutions had the following initial pH: 4.86 (2,4-DB), 4.14 (DCPP-P), 4.16 (MCPP-P), 4.19 (MCPA), and 6.64 (blank).

As previously mentioned, at low pH, pure HAs adsorb relatively large amounts of the neutral forms of PAAHs (which are much lower than the amounts adsorbed on FAs), whereas, at higher pH, only the anionic forms of MPCB and 2,4-DB are adsorbed. However, since HAs were complexed with Al^3+^, the anionic forms of MCPA, 2,4-D, MCPP-P, and DCPP-P were adsorbed in large amounts, whereas the adsorption of the anionic forms of MCPB and 2,4-DB was slightly inhibited [5]. It should be noted that within the pH range of 6.9–7.8, the addition of PAAHs to vigorously stirred suspensions of HAs complexed with Al^3+^ caused a notable increase in pH (Figure 3).

The adsorption of MCPB and 2,4-DB on HAs adjusted to pH 7.1 increased the right shoulder (3350–2900 cm^−1^) of the band (3423 cm^−1^). Moreover, a minor increase in the MCPB spectrum was noted around the band at 1242 cm^−1^. This suggested the existence of H-bonds with –OH groups of alcohols and phenols and hydrophobic interactions (Figure 2).

In the spectrum of 2,4-DB adsorbed on HAs+Al^3+^ at pH 7.1, the absorbance in the band at 3423 cm^−1^ slightly decreased, the changes in the range of 3350–2900 cm^−1^ disappeared, and minor absorbance decreasing was observed in the range of 1280–1100 cm^−1^. At 1593 cm^−1^, the bands for 2,4-DB were identical when this herbicide was adsorbed on pure HAs and in the HA+Al^3+^ system, which suggests that for both adsorbents, –COO^−^ groups were not involved in adsorption. It should be noted that in the HA+Al^3+^ system, only uncharged sorption sites associated with adsorbed and partially hydrolyzed Al^3+^ species were present on the surface of HAs at pH 7.1 [41].

The adsorption of DCPP-P and MCPA (representative derivatives of propionic acid and acetic acid, respectively) on HAs+Al^3+^ at pH 7.1 decreased absorbance of the band at 3423 cm^−1^ and increased the shoulder of the hydroxyl group band in the range of 3300–2900 cm^−1^, which suggests that H-bonds and hydrophobic interactions contributed to the binding of the anionic forms of these herbicides. Moreover, a minor increase was observed in the absorbance of the band at 1385 cm^−1^, which suggested that –COO^−^ groups in HAs with adsorbed Al^3+^ species made a small contribution to the adsorption of the anionic forms of these herbicides. 

### 2.2. Thermodynamic Analysis of the Adsorption of PAAH Anions on FAs+Al^3+^ and HAs+Al^3+^

#### 2.2.1. Thermodynamic Equilibrium Constant of Adsorption ($K_{0}$)

Thermodynamic parameters allow for a better understanding of the inherent energetic changes that occur during the adsorption processes and their underlying mechanisms. MCPA and DCPP-P were selected as adsorbates because our previous study [5] suggested that the anionic form of MCPA should have the lowest affinity for adsorption in humic substances via the bridges formed by the adsorbed Al^3+^ species, whereas the DCPP-P anions are characterized by one of the highest affinity. The adsorption experiments involving FA+Al^3+^ (pH 5.1) and HA+Al^3+^ (pH 8.0) suspensions were carried out at 5 °C, 20 °C, and 39 °C. 

The thermodynamic equilibrium constant ($K_{0}$), which is used to determine thermodynamic parameters, can be expressed by the following formula [42]:

(1) $$K_{0}=\frac{a_{s}}{a_{aq}}=\frac{\nu_{s}}{\nu_{aq}}\;\frac{C_{s}^{ads}(eq)}{C_{aq}^{ads}(eq)}$$

where $a_{s}$ and $a_{aq}$ denote the activities of the adsorbed PAAH anions and PAAH anions in the aqueous phase at the adsorption equilibrium; $\nu_{s}$ and $\nu_{aq}$ denote the activities of the adsorbed PAAH anions and PAAH anions in the aqueous phase; and $C_{s}^{ads}(eq)$ and $C_{aq}^{ads}(eq)$ denote the surface concentrations of PAAH anions (mmol/g) and the concentrations of PAAH anions in the solution at the adsorption equilibrium (mmol/mL), respectively. Equation (1) is reduced to the following form when the concentration of the solute in the solution approaches zero:

(2) $$K_{0}=\frac{a_{s}}{a_{aq}}=\frac{C_{s}^{ads}(eq)}{C_{aq}^{ads}(eq)}$$

The values of $K_{0}$ in Table 4 were estimated by plotting *l*$n\;\frac{C_{s}^{ads}(eq)}{C_{aq}^{ads}(eq)}$ versus $C_{aq}^{ads}(eq)$ and extrapolating $C_{s}^{ads}(eq)$ to zero [42].

The data presented in Table 4 indicate that at the same temperature, $K_{0}$ for adsorption of DCPP-P was higher than $K_{0}$ for MCPA adsorption, and that the adsorption for both herbicides decreased with a rise in temperature. Moreover, the adsorption of MCPA and DCPP-P was much higher on the FA+Al^3+^ fraction than on the HA+Al^3+^ fraction.

#### 2.2.2. Thermodynamic Parameters of Adsorption

The thermodynamic parameters of adsorption were determined based on the $K_{0}$ determined at three temperatures. The standard Gibbs free energy (Δ$G^{0}$) was calculated using the following equation [22,23]:

(3) $$\Delta G^{0}=-R\;T\ln K_{0}$$

where *R* is the molar gas constant (8.314 J/(mol K)), and *T* is absolute temperature. Next, ${\Delta H}^{0}$ and standard entropy (${\Delta S}^{0}$) were calculated from the plot $R\;lnK_{0}$ versus 1/T derived from the Van’t Hoff equation [22,23]:

(4) $$lnK_{0}=-\frac{\Delta H^{0}}{RT}+\frac{\Delta S^{0}}{R}$$

In this plot, the slope of the linear model obtained for three datapoints (1/T, $\mathrm{R ln}K_{0}$) is equal to ${-\Delta H}^{0}$ (J/mol) (see Equation (4)), and the intercept is equal to ${\Delta S}^{0}$ (J/(mol K)).

The obtained negative values of Δ$G^{0}$ (Table 4) indicate that the process is feasible and that PAAHs are spontaneously adsorbed on the surface of the examined adsorbents [42]. The obtained negative values of ${\Delta H}^{0}$ indicate that adsorption was an exothermic process. The values of ${\Delta H}^{0}$ values were always lower for MCPA and higher for DCPP-P, and they were lower for the FA+Al^3+^ fraction and higher for the HA+Al^3+^ fraction. Changes in adsorption enthalpy for physisorption and chemisorption generally occur in the range of −20 to 40 kJ/mol and −400 to −800 kJ/mol [22,23], respectively. Therefore, the obtained values in the range of −8.4 to −2.4 indicate that herbicide adsorption on the examined adsorbents was a physical process. Positive values of ${\Delta S}^{0}$ indicate randomness at the solid–solution interface during adsorption [22,23].

### 2.3. Molecular Modeling Results

**Figure 4:**
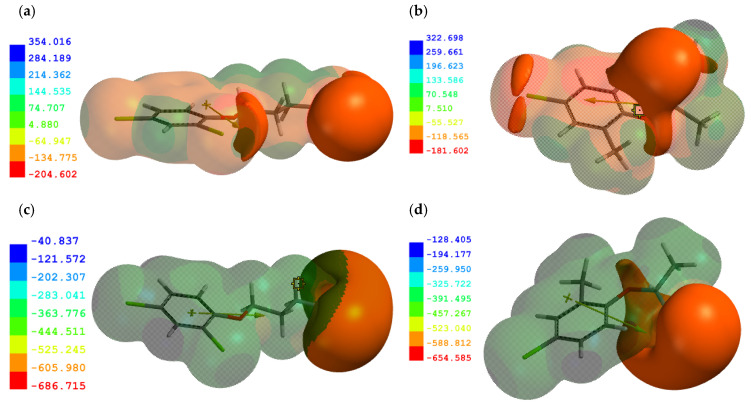
Molecular electrostatic potential (MEP) density of the molecules: (**a**) 2,4-DB; (**b**) MCPP-P and anionic forms: (**c**) 2,4-DB; (**d**) MCPP-P (kJ/mol), as well as their dipole moment. The electrostatic potential profile was superimposed on the MEPs at −83.7 kJ/mol for the neutral forms and −418.7 kJ/mol for the anionic forms. Deepest blue color—the highest positive potential; deepest red color—the highest negative potential; intermediate shades—intermediate potential regions.

The structure of the molecular and anionic forms of the examined PAAHs was analyzed in detail to determine the most probable mechanisms by which these PAAHs were adsorbed on FAs and HAs. The calculations were performed using the Hartree–Fock theory approach at 6-311G** level [43]), and it revealed that electrostatic charge distributions were similar for all PAAHs. Therefore, the neutral and anionic forms of 2,4-DB and MCPP-P characterized by the highest and lowest adsorption in soils [5] were regarded (Figure 4) as representatives in this study. Mulliken charges of atoms are presented in Table S1 in Supplementary Materials.

The highest negative potential was localized on the oxygen atom of phenoxy and carboxylic groups. In acetic acid derivatives, the negative potential formed a single area, whereas in butanoic and propionic acid derivatives, the negative potential was divided into two smallest areas around the –COOH group. A lower negative potential was also observed on Cl atoms, and it could play an important role in van der Waals interactions. In the anionic forms of PAAHs, negative potential increased significantly in the area of the –COO^−^ group (Figure 4c,d) (IzoValue at −418.7 kJ/mol). The dissociation process was also accompanied by a change in the value of the dipole moment.

## 3. Discussion—Adsorption Mechanisms Predicted Based on FTIR Data, Thermodynamic Parameters, and the Results of Molecular Modeling

### 3.1. Adsorption of the Neutral Forms of PAAHs

It is generally accepted that the neutral forms of acidic pesticides containing a carboxyl group form H-bonds with the involvement of these groups, as well as with the carboxyl, ester (R−COO−R’), or carbonyl (R−CO−R’) groups of organic matter [44,45]. H-bonds can also be formed by PAAHs, and they represent one of the key mechanisms by which PAAHs are adsorbed by humic substances. However, steric effects should also be considered because they can restrict the formation of H-bonds [44,45].

**Figure 5:**
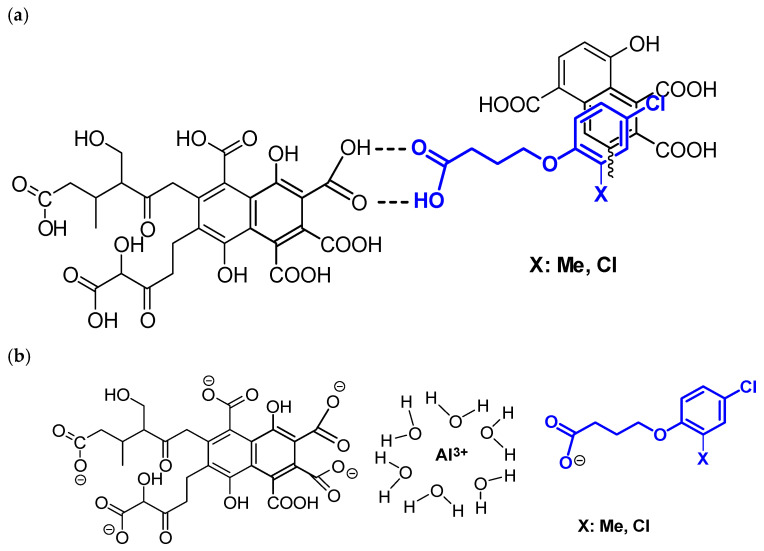
Proposed mechanisms of the adsorption of (**a**) the neutral forms of PAAHs at pH 2.9 on FAs and (**b**) the anionic forms of PAAHs at pH 5.7 on FAs complexed with Al^3+^. The model structure of FAs according to Buffle [46].

In the FTIR spectrum of FAs with adsorbed PAAHs (pH 2.9), changes were observed in the absorbance of broad bands formed by the groups involved in the formation of H-bonds, especially at 3427, 1718, and 1236 cm^−1^ (Figure 2). The most pronounced changes were noted for the most strongly adsorbed MCPB and 2,4-DB. In comparison with other acids, the adsorption of the molecular form of butanoic acid derivatives is much stronger because these compounds are weaker acids and contain the longest, unbranched alkyl chain. At pH 2.9, these compounds are characterized by the highest content of molecular forms that can form H-bonds and undergo adsorption on FAs. On the other hand, MCPB and 2,4-DB molecules have a significantly higher number of rotatable bonds (5, the remaining 3), which increases their conformational abilities and significantly reduces the requirements for the location of additional sorption sites of humic substances. It can be assumed that π-π stacking interactions between the phenyl ring and the aromatic structures of FAs are the second most important force. Due to the high lability of these molecules, such interactions are more likely to occur in butanoic acid derivatives, which contributes to an increase in their adsorption (Figure 5) relative to other PAAHs.

In addition, the non-polar surface and log P values increased in MCPB and 2,4-DB molecules, which promoted hydrophobic interactions (Table 1). Thus, the interactions between PAAHs with a long hydrocarbon chain and FAs are associated with an additional affinity for the hydrophobic areas of these molecules, which also contributes to an increase in their adsorption. This observation is supported by the fact that non-specific van der Waals forces were also highest in MCPB and 2,4-DB. These interactions decrease rapidly with an increase in the distance between the adsorbent and the adsorbate. Therefore, PAAH molecules with greater conformational ability, i.e., the ability to maintain close contact with the appropriate sites on the surface of the adsorbent [44], should make a greater contribution to the interactions in the adsorption process.

It can be assumed that these interactions played a key role in PAAHs that were adsorbed on HAs at pH 2.9. The share of these interactions changed only in response to changes in the structure of FAs and HAs. ^13^C CP/MAS NMR spectroscopy revealed more than a two-fold decrease in the number of -COOH groups in HAs (Table 2), which decreased the number of possible H-bonds. However, the FTIR spectra of HAs and ^13^C CP/MAS NMR data indicate that the share of aromatic fragments increased significantly, thus increasing the number of π-π stacking interactions, as well as their contribution to the adsorption forces in HAs. In turn, hydrophobic interactions were probably reduced due to the smaller share of alkyl fragments (Table 2).

Similar forces were described by Wu et al. [2] when MCPA was adsorbed at pH 2 on the hydrophobic acid (HOA) fraction of water-soluble organic matter with a similar structure to FAs. However, the contribution of hydrophobic interactions to total sorption on HOA was higher in the cited study. Wu et al. [2] argued that the aromatic structure associated with the polar groups of organic matter polar fractions plays a key role in the interactions with phenoxy herbicides at low pH, which corroborates the results of the present study.

An analysis of the pKa values of PAAHs (Table 1) and the pH range of arable soils indicates that the above adsorption mechanisms should play a dominant role in the retention of MCPB and 2,4-DB in soils, as well as their transport, in the form of adsorbates with water-soluble humic substances, to groundwater and surface water. For other PAAHs, the extent of their retention in soil and transport to groundwater and surface water is determined by the adsorption mechanisms of their anionic forms.

In conclusion, the differences in the acidity (pKa) of butanoic, propanoic, and acetic acid derivatives, the differences in log P values, and the differences in the lability of the examined PAAHs were responsible for the differences in the adsorption of their molecular forms on FAs and HAs.

### 3.2. Adsorption of Al^3+^ and the Anionic Forms of PAAHs

The addition of Al^3+^ species to the solution led to the coagulation of FA colloids, with a maximum at pH 5.0−5.5 [47]. Changes in the absorbance of the bands corresponding to asymmetric and symmetric stretching of –COO^−^ groups were noted in the FTIR spectrum after the addition of Al^3+^ (at pH 5.7 in FAs and at pH 7.1 in HAs), but shifts were not observed. The above can be attributed to the formation of outer-sphere complexes between –COO^−^ and Al^3+^ species [31]. Based on the observation that at pH 5.7, Al^3+^ species occur in an aqueous solution in the following sequence: Al(OH)_2_^+^ > Al(OH)_3_ > AlOH^2+^ > Al^3+^ [41], and the assumption that the adsorption of Al^3+^ on FAs neither favors nor inhibits hydrolysis [36], and that bidentate complexes composed of two –COO^−^ groups are predominant [48], it can be hypothesized that [(–COO^−^)_2_Al^3+^(H_2_O)_6_]^+^ and [(–COO^−^)_2_Al^3+^(H_2_O)_5_OH^−^] [49] were the predominant outer-sphere complexes. Numerous authors [36,47,50] have postulated that Al^3+^ is complexed by humic substances in partially hydrolyzed forms. Research has shown that outer- and intra-sphere complexes can be formed between humic substances and Al^3+^, and a predominance of the former was reported by Nordin et al. [51], Guan et al. [52], and Elkins et al. [18], whereas a predominance of the latter was postulated by Aquino et al. [49] and Jin et al. [31]. In studies analyzing the thermodynamics of the interactions between Al^3+^ and humic substances, the values of ${\Delta H}^{0}$ ranged from −10.52 kJ/mol to 2.19 kJ/mol [53,54], i.e., were within the physical sorption range [22,23], which suggests that outer-sphere complexes should predominate [40]. It should also be noted that bidentate Al^3+^–humic substance complexes can be formed by carboxyl groups forming an oxalic acid-like ligand [47], carboxyl groups of the adjacent hydrocarbon chains [49]), and, above all, two carboxyl groups located in the 1,2 position of the benzene ring [55].

At pH 5.7, the absorbance of the band corresponding to –OH groups in FAs (3425–3431 cm^−1^) after the addition of Al^3+^ was higher than the absorbance of the band corresponding to pure FAs (Figure 1a). This difference can be largely attributed to the superposition of the vibrations of –OH groups in FAs with the vibrations of –OH groups in the adsorbed and precipitated Al(OH)_3_ [56]. In turn, the absorbance of the pure FAs band was lower at pH 5.7 than at pH 2.9, probably due to the superposition of OH groups in –COOH that were not dissociated at pH 2.9. However, these results do not rule out the presence of H-bonds, including the bonds between the hydroxyl group of the [(–COO^−^)_2_Al^3+^(H_2_O)_5_OH^−^] complex and the adjacent hydroxyl or phenolic groups of FAs or HAs. The presence of such an adsorption mechanism has been suggested by Lu et al. [47] and Jin et al. [31].

As previously mentioned, the maximum adsorption of anionic forms of PAAHs on FAs complexed with Al^3+^ occurred at around pH 5.0–5.5, and adsorption decreased to zero at pH~7.0 [5]. The above suggests that adsorption decreased with a decline in the number of positive sorption sites of the adsorbed Al^3+^ species. Assuming that outer-sphere complexes [(–COO^−^)_2_Al^3+^(H_2_O)_6_]^+^ were the dominant type of positive sorption sites in the studied FAs, and given that the ${\Delta H}^{0}$ values for the adsorption of the anionic forms of PAAHs (denoted as [PAA]¯) ranged from −8.4 kJ/mol to −2.9 kJ/mol (which is why adsorption was not associated with the band shifts of their –COO¯ groups complexed with Al^3+^), the dominant adsorption mechanism can be described by a very simple equation:

[(–COO^−^)_2_Al^3+^(H_2_O)_6_]^+^ + [PAA]^−^ ⇔ [(–COO^−^)_2_Al^3+^(H_2_O)_6_]^+^[PAA]^−^ (5)

Moreover, visible changes in band absorbance at 3431, 1238, and 1003 cm^−1^ (Figure 2a) point to the presence of H-bonds between the ether group of the PAAH anion and the hydrogen atom of the hydroxyl or phenol group, or even the hydroxyl group of polysaccharides. In addition, changes in absorbance at 2976 and 2930 cm^−1^ point to the presence of hydrophobic interactions between the aliphatic chains of PAAHs and FAs.

At pH 7.1, pure HAs adsorbed only MCPB and 2,4-DB anions. The adsorption process occurred at this pH despite the fact that the dissociated carboxyl groups of the adsorbate and adsorbent repelled each other. The changes in bands absorbance at 3350–2900 cm^−1^ and at ~1242 cm^−1^ indicate that adsorption was mediated by hydrophobic interactions between the aliphatic chains of the adsorbate and the adsorbent and by H-bonds with alcohols and phenols [38]. However, the large number of aromatic groups in HAs and the small number of hydrocarbon chains suggest that π-π stacking interactions were dominant. The fact that only MCPB and 2,4-DB anions were adsorbed can be attributed to their structure—these PAAHs have the longest unbranched hydrocarbon chain with large conformational possibilities (highest flexibility) and are characterized by the highest log P values.

The adsorption of the anionic forms of MCPB and 2,4-DB decreased considerably after the formation of HA+Al^3+^ complexes. A comparison of the FTIR spectrum of the adsorbent with the spectra after the adsorption of MCPB and 2,4-DB anions indicates that only H-bonds participated in the adsorption of herbicides (significant changes in absorbance were observed at 3423 cm^−1^ and minor changes were noted at 1280–1100 cm^−1^). In contrast, the formation of HA+Al^3+^ complexes led to the adsorption of the anionic forms of 2,4-D, MCPA, DCPP-P, and MCPP-P. In this case, changes in band absorbance were observed at 3423 cm^−1^ and in the range of 3300–2900 cm^−1^ (H-bonds and hydrophobic interactions), and at 1385 cm^−1^ (interactions with carboxyl anions with adsorbed Al^3+^). However, it seems that also in this case the π-π stacking interactions should occur.

As previously noted, the adsorption of the anionic forms of all PAAHs by HAs complexed with Al^3+^ was independent of pH (the same adsorption was observed for individual herbicides in the pH range of 5.5–8.0). Experiments (Figure 3) showed that the addition of the anionic form of PAAHs to the suspension of HAs complexed with Al^3+^ caused an immediate increase in pH. Assuming that only [(–COO¯)_2_AlOH^2+^(H_2_O)_5_] and [(─COO¯)_2_Al(OH)_2_^+^(H_2_O)_4_]¯ complexes were present on the surface of HAs in the pH range of 7.0–8.0, it can be hypothesized that the addition of the anionic form of PAAHs caused the following reaction to occur:

[(–COO^−^)_2_AlOH^2+^(H_2_O)_5_] + [PAA]^−^ + H_2_O ⇔ [(–COO^¯^)_2_Al^3+^(H_2_O)_6_]^+^ [PAA]^−^ + OH^−^ (6)

**Figure 6:**
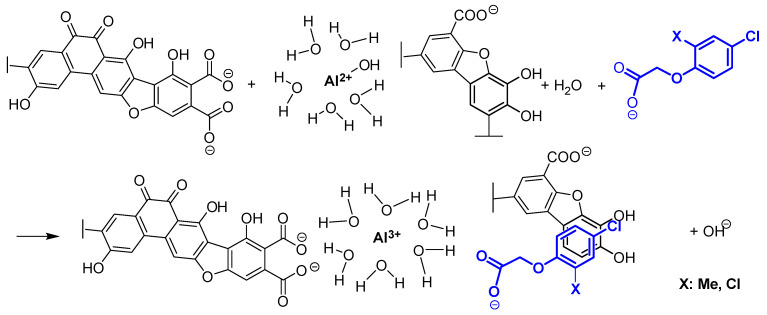
Proposed mechanisms of the adsorption of the anionic forms of PAAHs at pH 7.1 on HAs complexed with Al^3+^. The model structure of HAs according to Stevenson [57].

It should be assumed that reaction (6) occurred simultaneously with sufficiently strong H-bonds supported by hydrophobic and/or π-π interactions (Figure 6). If H-bonds, hydrophobic, and π-π interactions were the dominant forces in the adsorption process, and the ligand exchange mechanism was only an accompanying process, the proposed mechanism could occur in the pH range of ~5.5–8.0, independently of pH. Moreover, this mechanism explains the observed changes in pH (Figure 3); it is consistent with the obtained FTIR results (Figure 2), the differences in the structure of FAs and HAs (Table 2), and ${\Delta H}^{0}$ values (Table 4).

## 4. Materials and Methods

### 4.1. Materials and Chemicals

Methanol stock solutions (250 mg/L) of the analyzed PAAHs were prepared using certified analytical standards (99.9% ± 0.1, Sigma-Aldrich, Poznań, Poland; or 99.7–99.8% ± 0.1, Institute of Organic Industrial Chemistry, Warsaw, Poland) and HPLC-grade methanol. Aqueous solutions of PAAHs (25 mg/L), were prepared from the stock solutions using ultrapure water with a conductivity of 0.05 µS/cm. The remaining chemicals and solvents used in this study were of analytical or HPLC grade.

### 4.2. Isolation of FAs and HAs

The examined FA fraction was isolated from the Ap horizon of typical Polish Luvisols (OC content 0.88%, pH 4.5), and the HA fraction was isolated from the Ap horizon of typical Chernozems (OC of 1.92%, pH 7.1) [58] (classification according to WRB [59]). These soils have been assigned code numbers 590 and 587, respectively, in the Database of Polish Arable Mineral Soils [58] kept by the Institute of Agrophysics of the Polish Academy of Sciences in Lublin. The properties and locations of the above soil profiles were presented previously by Siek and Paszko [60]. FAs and HA fractions were extracted using 0.1 M Na_4_P_2_O_7_, and the obtained extracts were acidified to pH 1.2 with 6 M HCl to promote the sedimentation of HAs [13,14,61]. The FA fraction was isolated with Amberlite^TM^ XAD7HP resin from Supelco^®^ (Saint Louis, MO, USA) [61], and the HA fraction was purified according to the IHSS [14] procedure. The FA and HA isolation procedures were described in detail by Paszko et al. [5].

### 4.3. Adsorption Experiments

#### 4.3.1. Adsorption of PAAHs on FAs at pH 2.9 and FAs+Al^3+^ at pH 5.7

A stock solution of FAs (0.468 mg/mL) was used in adsorption experiments. In the experiments conducted at native pH, the stock FA solution was pipetted (7.0 mL) into duplicate test tubes, followed by 0.4 M KCl solution (0.677 mL), and 25 mg/L of the PAAH solution (5.86 mL) or the same volume of ultrapure water with methanol (9:1 *v*/*v*; blank sample). The test tubes were agitated for 24 h on a rotator (20 rpm/min) at 20 ± 0.5 °C; the pH of the solutions in the test tubes was measured at the end of agitation, and the duplicate samples were freeze-dried for the FTIR analysis. The initial concentration of PAAHs in the FA solution was 10.8 mg/L (the OC ratio of FAs to PAAHs was 10:1 mg/mg), and the concentration of KCl (inert electrolyte) was 0.02 M.

In adsorption experiments conducted at pH 5.7 using FA+Al^3+^ suspensions, the stock FA solution (7.0 mL) and 0.1 M KOH solution were pipetted (0.63 mL; pH was increased to ~5) into duplicate test tubes, and the tubes were agitated (0.5 h). Next, 3 mg/mL of Al^3+^ was pipetted (0.685 mL; AlCl_3_ was used); the test tubes were agitated (0.5 h); 0.1 M KOH solution was added (1.54 mL), and the tubes were agitated again (0.5 h). Finally, 0.4 M KCl solution (0.827 mL) and 25 mg/L of the PAAH solution (5.86 mL) or the same volume of the blank sample were pipetted into the test tubes. The following steps were identical to those applied to pure FAs. The initial concentration of PAAHs in the FA+Al^3+^ suspension was 8.86 mg/L (the OC ratio of FAs to PAAHs was 10:1 mg/mg); the concentration of KCl was 0.02 M, and the mean Al^3+^–PAAH ratio was 117:1 mmol/mmol.

In analyses investigating the thermodynamic parameters for MCPA and DCPP-P adsorption at pH 5.1 on FAs complexed with Al^3+^, the stock FA solution was pipetted (0.800 mL) into test tubes and combined with ultrapure water (0.325 mL). Next, 0.1 M NaOH was added (0.070 mL) to adjust the pH to ~5.2, and the samples were agitated on a rotator (1 h, 20 rpm, 20 °C); 4.94 mg/mL of AlCl_3_ (0.117 mL) was added, and the samples were agitated (1 h); the pH was once again adjusted to ~5.2 by adding 0.1 M NaOH (0.100 mL) and the samples were agitated (1 h); again 4.94 mg/mL of AlCl_3_ (0.117 mL) was added and again the samples were agitated (1 h); 0.1 M NaOH (0.117 mL) and 0.4 M CaCl_2_ (0.045 mL) were added, and the samples were agitated again (12 h, 20 rpm, 20 °C). In the following step, the pH of each of the 36 samples was measured, and a third of the samples were agitated (2 h) at 5 °C, 20 °C, or 39 °C. Next, 5 mg/L, 10 mg/L, or 25 mg/L solutions of MCPA or DCPP-P were added in duplicate (0.109 mL), the samples were agitated at 5 °C, 20 °C, or 39 °C (24 h, 20 rpm), centrifuged at 5 °C, 20 °C, or 39 °C (20 min, 3300× *g*), and the liquid phase was sampled for HPLC analysis. A detailed description of the HPLC analysis can be found in [5].

#### 4.3.2. Adsorption of PAAHs on HAs, and HAs+Al^3+^ at pH 7.1

The adsorption of PAAHs on pure HAs at pH 7.1 was examined using duplicate HA samples (3.5 mg) combined with ultrapure water (1.265 mL) and 0.1 M KOH (0.190 mL). The suspensions in the test tubes were agitated on a rotator (0.5 h); 0.4 M KCl solution was added (0.09 mL), followed by solutions (0.252 mL) of 25 mg/L of MCPB or 2,4-DB or the same volume of a blank sample. The initial concentration of PAAHs in 0.02 M KCl was 3.5 mg/mL, and the OC ratio of HAs to PAAHs was 300:1 mg/mg. The test tubes were agitated on a rotator (24 h); the pH of HA suspensions was measured at the end of agitation, and the samples were freeze-dried for the FTIR analysis.

The adsorption of PAAHs on HAs with the adsorbed Al^3+^ species at pH 7.1 was examined using the duplicate HA samples (3.5 mg) combined with ultrapure water (0.848 mL) and 0.1 M KOH (0.100 mL; pH was increased to ~5). The suspensions in the test tubes were agitated (0.5 h); 0.3 mg/mL of the Al^3+^ solution (as AlCl_3_) was added (0.35 mL); the test tubes were agitated again (0.5 h); 0.4 M KCl (0.09 mL) and 0.1 M KOH (0.100 mL; pH was increased to ~7.1) were added, and the tubes were agitated for the third time (2.0 h). Finally, 25 mg/L solutions of selected PAAHs or the blank sample (0.252 mL) were added. The following steps, including agitation for 24 h, were identical to those applied to pure HAs.

The above procedure was used in separate experiments analyzing changes in pH during the adsorption of PAAHs on HAs complexed with Al^3+^ species, but HA suspensions were stirred rapidly using a magnetic stirrer. After the addition of 0.4 M KCl (0.09 mL), 0.1 M KOH was added to adjust pH to 6.9–7.8, and the pH of the stirred suspension was measured at 1 min intervals during equilibration (3.0 h). Next, 25 mg/L solution (0.252 mL) of selected PAAHs was added, the pH was measured, and the suspension was equilibrated (1.0 h). The results, limited to 4 min before and 6 min after the addition of PAAH or the blank solution, are presented in Figure 3.

In experiments analyzing the thermodynamic parameters of MCPA and DCPP-P adsorption at pH 8 on HAs complexed with Al^3+^, 36 HA samples were weighed (2.9 mg) into test tubes, ultrapure water was added (1.028 mL), pH was adjusted to ~5.2 with 0.1 M NaOH (0.081 mL), and the samples were agitated ((1 h, 20 rpm, 20 °C). Next, 1.65 mg/mL of AlCl_3_ was added (0.282 mL), and the samples were agitated (1 h); 0.1 M NaOH (0.214 mL) and 0.4 M CaCl_2_ (0.045 mL) were added, and the samples were agitated again (12 h, 20 rpm, 20 °C). In the following step, the pH of the samples was measured and a third of the samples were agitated (2 h) at 5 °C, 20 °C, or 39 °C. Next 3 mg/L, 6 mg/L, or 9 mg/L solutions of MCPA or DCPP-P were added in duplicate (0.150 mL); the samples were agitated at 5 °C, 20 °C, or 39 °C (24 h, 20 rpm), were centrifuged at 5 °C, 20 °C, or 39 °C (20 min, 3300× *g*), and the liquid phase was sampled for HPLC analysis.

### 4.4. Fourier-Transform Infrared Spectroscopy

Fourier-transform infrared spectroscopy was used to assess the potential interactions between FAs or HAs, the added Al^3+^, and PAAHs. For this purpose, 1 mg of freeze-dried preparations were combined with 200 mg of spectrally pure KCl to obtain a homogeneous mixture that was pressed into thin pellets (10 tons in 5 min). FTIR spectra were recorded on a Tensor 27 FTIR spectrometer (Bruker Optics, Billerica, MA, USA) in the range of 900–3700 cm^−1^, with a spectral resolution of 2 cm^−1^, the number of scans was 256 items. Raw data were processed with a 25-point smoothing function based on the Savitsky–Golay algorithm to reduce spectral noise and produce more meaningful spectra. Data were converted from absorbance to transmittance using the absorbance = 2-log(transmittance) formula, and the results were corrected for atmospheric CO_2_. The baseline was corrected using the rubberband method (100 baseline points, 50 iterations), and the entire spectral range was normalized with a vector technique.

### 4.5. Molecular Modeling

The models of PAAH molecules and anions were built with standard bond lengths and angles using the PC SPARATN’10 Pro Ver. 1.1.0 molecular modeling software [62]. The energy was minimized by molecular mechanical methods, and the conformer with the lowest potential energy was used in further analyses. The structure was optimized with the Hartree–Fock method at 6-311+G** level and equilibrium geometry of the ground state [62]. In ab initio calculations, 6-311+G** is a valence triple-zeta polarized basis set that adds a set of polarizing d-functions to heavy atoms and a set of polarization p-functions to hydrogen (6-311+G(d,p)) [43]. It is often applied to calculate the electronic properties of small organic molecules with biological potency [63,64,65]. The Mulliken atomic charge was determined according to Singh and Kollman [66]. The calculations for anionic forms were conducted based on the total anion charge.

## 5. Conclusions

This study demonstrated that the adsorption mechanisms of PAAHs on pure FAs and HAs and on FAs and HAs complexed with Al^3+^ involved physical adsorption mechanisms. This observation was confirmed by the obtained thermodynamic parameters of adsorption and FTIR spectra.

At low pH, the neutral forms of PAAHs were bound mainly via H-bonds between their non-dissociated carboxyl groups and the carboxyl or hydroxyl groups of FAs and HAs. Moreover, the presence of hydrophobic and π-π stacking interactions with FAs and HAs was observed, mostly in the case of MCPB and 2,4-DB molecules. These derivatives of butyric acid have five rotatable bonds (the remaining ones have three), which significantly increases their conformational abilities.

The highest conformational abilities of MCPB and 2,4-DB also promoted the adsorption of their anionic forms on surface HAs at high pH, despite the fact that HA surfaces were partly negatively charged due to the dissociation of their carboxyl groups. The anionic forms of MCPB and 2,4-DB were adsorbed on HAs mainly via hydrophobic and π-π stacking interactions and H-bonds.

The complexation of FAs and HAs with Al^3+^ created a positive charge on their surfaces, mainly due to the formation of Al^3+^ bridges with two dissociated carboxyl groups of FAs and HAs. Both types of humic substances formed outer-sphere complexes with Al^3+^. The complexation of FAs with Al^3+^ promoted the formation of outer-sphere complexes between the anionic forms of PAAHs and the outer-sphere complexes of FAs with Al^3+^. In the created PAAH–Al^3+^–FA ternary complex, the electrostatic interactions between the ─COO^−^ group of PAAHs, [Al(H_2_O)_6_]^3+^, and two –COO^−^ groups of FAs were dominant. The above complex should exist at a pH up to 7–7.5 because at pH 7–7.5, only Al(OH)_3_ and small amounts of [Al(OH)_4_]^+^ exist in aquatic solution.

The complexation with Al^3+^ led to the coagulation of FAs and HAs, which largely decreased the surface of these complexes and, consequently, the number of their available sorption sites. This is the main reason why the adsorption of the anionic forms of MCPB and 2,4-DB was lower on HAs+Al^3+^ than on pure HAs (none of the anionic forms of PAAHs was adsorbed on pure FAs, and only the anionic forms of MCPB and 2,4-DB on pure HAs).

Contrary to adsorption on FAs+Al^3+^, the adsorption of the anionic forms of PAAHs was independent of pH and occurred even at pH > 8. The study demonstrated that the adsorption of the anionic forms of PAAHs on HAs+Al^3+^ was associated with a slight increase in pH. Therefore, it was postulated that the mechanisms of adsorption on HAs involved a ligand exchange between a loosely bound hydroxyl group of hydrolyzed Al^3+^ complexed with this adsorbent and the anionic form of the herbicide. However, in this case, adsorption took place only in the presence of sufficiently strong hydrophobic and π-π stacking interactions supported by H-bonds, and the ligand exchange mechanism was only an accompanying process. When the above conditions are fulfilled, the proposed mechanism could occur in the pH range of ~5.5–8.0, independently of pH.

The finding that PAAHs in soil are primarily bound to FAs, and to a lesser extent, to HAs via physical adsorption mechanisms has practical implications. It helps understand why modeling of the movement of PAAHs through the soil profile has generally shown that these compounds should not accumulate in the soil profile and move to groundwater at concentrations exceeding the permissible concentration, while the results of monitoring studies have shown their concentrations > 0.1 μg/L. The reason for this discrepancy is the adsorption of a large part of PAAHs by the FA fraction, followed by the transport of part of this fraction to groundwater and surface water. Physical adsorption processes are strong enough to prevent PAAH degradation by microorganisms since microbial degradation occurs predominantly in the aqueous phase. On the other hand, these processes are reversible, and desorption can easily occur under favorable conditions.

## Figures and Tables

**Table 2:** Results of the elemental analysis and integrals (%) of ^13^C CP/MAS NMR spectra of the examined humic substances. Refer to [5] for detailed information.

Element/Assignment	FAs (%)	HAs (%)
C	44.72	53.94
H	4.65	3.19
N	3.25	3.15
S	0.61	0.26
Aliphatic (0–110 ppm)	30.2	8.7
Ar (110–157 ppm)	35.1	50.4
CH_3_ (0–43 ppm)	7.3	4.3
OCH_3_ (45–60 ppm)	5.2	1.5
Side-chain lignin (43–87 ppm)	15.7	3.0
Ar-OH (145–165 ppm)	Nd ^1^	Nd
COOH (158–190 ppm)	41.8	18.0
HB ^1^	0.90	2.81

Nd—not determined. ^1^ Hydrophobicity, determined as follows [32]: HB = [(0–43) + (110–160)]/[(45–60) + (145–190)].

**Table 4:** Thermodynamic parameters of the adsorption of MCPA and DCPP-P on FAs and HAs complexed with Al^3+^ species.

Sample	PAAH	pH	T(K)	$K_{0}$	$\Delta G^{0}$(kJ/mol)	$\Delta H^{0}$(kJ/mol)	$\Delta S^{0}$(J/(mol K))	R^2 1^
FAs+Al^3+^	MCPA	5.10	278.15	267.00	−12.92	−8.4	16.2	0.985
			293.15	231.49	−13.27			
			312.15	179.74	−13.47			
FAs+Al^3+^	DCPP-P	5.08	278.15	450.18	−14.13	−2.9	40.3	0.973
			293.15	413.76	−14.69			
			312.15	392.13	−15.50			
HAs+Al^3+^	MCPA	7.97	278.15	36.91	−8.34	−5.3	10.8	0.907
			293.15	30.59	−8.34			
			312.15	28.68	−8.71			
HAs+Al^3+^	DCPP-P	7.96	278.15	58.86	−9.42	−2.4	25.5	0.958
			293.15	57.03	−9.86			
			312.15	52.73	−10.29			

^1^ Coefficient of determination.

## Data Availability

All relevant data are within the text and the Supplementary Materials.

## Supplementary Materials

The following supporting information can be downloaded at https://www.mdpi.com/article/10.3390/ijms252312699/s1.

## References

[1] Zimmermán P.W., Hitchcock A.E. (1942). Substituted phenoxy and benzoic acid growth substances and the relation of structure to physiological activity. Contrib. Boyce Thomps..

[2] Wu D.M., Ren C.Q., Jiang L., Li Q.F., Zhang W., Wu C.Y. (2020). Characteristic of dissolved organic matter polar fractions with variable sources by spectrum technologies: Chemical properties and interaction with phenoxy herbicide. Sci. Total Environ..

[3] Loos R., Locoro G., Comero S., Contini S., Schwesig D., Werres F., Balsaa P., Gans O., Weiss S., Blaha L. (2010). Pan-European survey on the occurrence of selected polar organic persistent pollutants in ground water. Water Res..

[4] Loos R., Gawlik B.M., Locoro G., Rimaviciute E., Contini S., Bidoglio G. (2009). EU-wide survey of polar organic persistent pollutants in European river waters. Environ. Pollut..

[5] Paszko T., Spadotto C.A., Huber M., Jerzykiewicz M., Matysiak J., Skrzypek A., Boguta P. (2024). Can the pH-dependent adsorption of phenoxyalkanoic herbicides in soils be described with a single equation?. Environ. Sci. Pollut. Res..

[6] Spadotto C.A., Hornsby A.G. (2003). Soil sorption of acidic pesticides: Modeling pH effects. J. Environ. Qual..

[7] Kah M., Brown C.D. (2007). Prediction of the adsorption of ionizable pesticides in soils. J. Agric. Food Chem..

[8] Paszko T., Muszyński P., Materska M., Bojanowska M., Kostecka M., Jackowska I. (2016). Adsorption and degradation of phenoxyalkanoic acid herbicides in soils: A review. Environ. Toxicol. Chem..

[9] Lewis K.A., Tzilivakis J., Warner D.J., Green A. (2016). An international database for pesticide risk assessments and management. Hum. Ecol. Risk Assess..

[10] Shariff R.M. (2011). Thermodynamic adsorption-desorption of metolachlor and 2,4-D on agricultural soils. Int. J. Chem..

[11] Nakashima K., Xing S.Y., Gong Y.K., Miyajima T. (2008). Characterization of humic acids by two-dimensional correlation fluorescence spectroscopy. J. Mol. Str..

[12] Iglesias A., López R., Gondar D., Antelo J., Fiol S., Arce F. (2009). Effect of pH and ionic strength on the binding of paraquat and MCPA by soil fulvic and humic acids. Chemosphere.

[13] Audette Y., Longstaffe J.G., Gillespie A.W., Smith D.S., Voroney R.P. (2021). Validation and comparisons of NaOH and Na_3_PO_4_ extraction methods for the characterization of organic amendments. Soil Sci. Soc. Am. J..

[14] IHSS (2024). Isolation of IHSS Soil Fulvic and Humic Acids. https://humic-substances.org/isolation-of-ihss-soil-fulvic-and-humic-acids/.

[15] Khan S.U. (1973). Equilibrium and kinetic studies of adsorption of 2,4-D and picloram on humic acid. Can. J. Soil Sci..

[16] Celis R., Hermosin M.C., Cox L., Cornejo J. (1999). Sorption of 2,4-dichlorophenoxyacetic acid by model particles simulating naturally occurring soil colloids. Environ. Sci. Technol..

[17] Haberhauer G., Temmel B., Gerzabek M.H. (2002). Influence of dissolved humic substances on the leaching of MCPA in a soil column experiment. Chemosphere.

[18] Elkins K.M., Dickerson M.A., Traudt E.M. (2011). Fluorescence characterization of the interaction Suwannee river fulvic acid with the herbicide dichlorprop (2-(2,4-dichlorophenoxy)propionic acid) in the absence and presence of aluminum or erbium. J. Inorg. Biochem..

[19] Ćwieląg-Piasecka I., Medyńska-Juraszek A., Jerzykiewicz M., Dębicka M., Bekier J., Jamroz E., Kawałko D. (2018). Humic acid and biochar as specific sorbents of pesticides. J. Soil. Sediments.

[20] Haberhauer G., Pfeiffer L., Gerzabek M.H. (2000). Influence of molecular structure on sorption of phenoxyalkanoic herbicides on soil and its particle size fractions. J. Agric. Food Chem..

[21] Kah M., Brown C.D. (2006). Adsorption of ionisable pesticides in soils. Rev. Environ. Contam. Toxicol..

[22] Khan M.A., Kim S.-w., Rao R.A.K., Abou-Shanab R.A.I., Bhatnagar A. (2010). Adsorption studies of dichloromethane on some commercially available GACs: Effect of kinetics, thermodynamics and competitive ions. J. Hazards Mater..

[23] Lian L., Guo L., Guo C. (2009). Adsorption of Congo red from aqueous solutions onto Ca-bentonite. J. Hazards Mater..

[24] European Commision (2024). EU Pesticides Database. https://ec.europa.eu/food/plants/pesticides/eu-pesticides-database_en.

[25] Albert A., Serjeant E.P. (1984). The Determination of Ionization Constants. A Laboratory Manual.

[26] Leo A.J. (1991). Calculating the hydrophobicity of chlorinated hydrocarbon solutes. Sci. Total Environ..

[27] Leo A.J. (1993). Calculating Log P_oct_ from structures. Chem. Rev..

[28] Sannino F., Violante A., Gianfreda L. (1997). Adsorption-desorption of 2,4-D by hydroxy aluminium montmorillonite complexes. Pestic. Sci..

[29] Piccolo A., Stevenson F.J. (1982). Infrared-spectra of Cu^2+^, Pb^2+^, and Ca^2+^ complexes of soil humic substances. Geoderma.

[30] Larrivee E.M., Elkins K.M., Andrews S.E., Nelson D.J. (2003). Fluorescence characterization of the interaction of Al and Pd with Suwannee River fulvic acid in the absence and presence of the herbicide 2,4-dichlorophenoxyacetic acid. J. Inorg. Biochem..

[31] Jin P.K., Song J.N., Wang X.C.C., Jin X. (2018). Two-dimensional correlation spectroscopic analysis on the interaction between humic acids and aluminum coagulant. J. Environ. Sci..

[32] Xu J.S., Zhao B.Z., Chu W.Y., Mao J.D., Olk D.C., Xin X.L., Zhang J.B. (2017). Altered humin compositions under organic and inorganic fertilization on an intensively cultivated sandy loam soil. Sci. Total Environ..

[33] Wu M., Song M., Liu M., Jiang C., Li Z. (2016). Fungicidal activities of soil humic/fulvic acids related to their chemical structures in greenhouse vegetable fields with cultivation chronosequence. Sci. Rep..

[34] Plaza C., Senesi N., Polo A., Brunetti G., García-Gil J.C., D’Orazio V. (2003). Soil fulvic acid properties as a means to assess the use of pig slurry amendment. Soil Till. Res..

[35] Cavoski I., D’Orazio V., Miano T. (2009). Interactions between rotenone and humic acids by means of FT-IR and fluorescence spectroscopies. Anal. Bioanal. Chem..

[36] Simonsson M. (2000). Interactions of aluminium and fulvic acid in moderately acid solutions: Stoichiometry of the H^+^/Al^3+^ exchange. Eur. J. Soil Sci..

[37] Boguta P., D’Orazio V., Senesi N., Sokolowska Z., Szewczuk-Karpisz K. (2019). Insight into the interaction mechanism of iron ions with soil humic acids. The effect of the pH and chemical properties of humic acids. J. Environ. Manag..

[38] Silverstein R.M., Webster F.X., Kiemle D.J. (2005). Spectrometric Identification of Organic Compounds.

[39] Zhang Z.Q., Gao Q., Xie Z.L., Yang J.M., Liu J.H. (2021). Adsorption of nitrification inhibitor nitrapyrin by humic acid and fulvic acid in black soil: Characteristics and mechanism. RSC Adv..

[40] Sposito G. (1989). The Chemistry of Soils.

[41] Gensemer R.W., Playle R.C. (1999). The bioavailability and toxicity of aluminum in aquatic environments. Crit. Rev. Environ. Sci. Technol..

[42] Zhang J.Y., Wu C.D., Jia A.Y., Hu B. (2014). Kinetics, equilibrium and thermodynamics of the sorption of *p*-nitrophenol on two variable charge soils of Southern China. Appl. Surf. Sci..

[43] Reyes A., Moncada F., Charry J. (2018). The any particle molecular orbital approach: A short review of the theory and applications. Int. J. Quantum Chem..

[44] Senesi N. (1992). Binding mechanisms of pesticides to soil humic substances. Sci. Total Environ..

[45] Barchańska H., Czaplicka M., Kyzioł-Komosińska J. (2020). Interaction of selected pesticides with mineral and organic soil components. Arch. Environ. Prot..

[46] Buffle J.A.E. (1977). Les substances humiques et leurs interactions avec les ions mineraux. Conference Proceedings de la Commisison d’Hydrologie Appliquee de l’A.G.H.T.M..

[47] Lu X.Q., Chen Z.L., Yang X.H. (1999). Spectroscopic study of aluminium speciation in removing humic substances by Al coagulation. Water Res..

[48] Nissinen A., Ilvesniemi H., Tanskanen N. (1999). Equilibria of weak acids and organic Al complexes explain activity of H and Al in a salt extract of exchangeable cations. Eur. J. Soil Sci..

[49] Aquino A.J.A., Tunega D., Schaumann G.E., Haberhauer G., Gerzabek M.H., Lischka H. (2014). Proton transfer processes in polar regions of humic substances initiated by aqueous aluminum cation bridges: A computational study. Geoderma.

[50] Shin J.Y., Spinette R.F., O’Melia C.R. (2008). Stoichiometry of coagulation revisited. Environ. Sci. Technol..

[51] Nordin J., Persson P., Laiti E., Sjoberg S. (1997). Adsorption of o-phthalate at the water-boehmite (gamma-AlOOH) interface: Evidence for two coordination modes. Langmuir.

[52] Guan X.H., Shang C., Chen G.H. (2006). ATR-FTIR investigation of the role of phenolic groups in the interaction of some NOM model compounds with aluminum hydroxide. Chemosphere.

[53] Zhao X., Wang T., Du G., Zheng M., Liu S., Zhang Z., Zhang Y., Gao X., Gao Z. (2019). Effective removal of humic acid from aqueous solution in an Al-based metal-organic framework. J. Chem. Eng. Data.

[54] Bryan N.D., Hesketh N., Livens F.R., Tipping E., Jones M.N. (1998). Metal ion-humic interaction. A thermodynamic study. J. Chem. Soc. Faraday Trans..

[55] McBride M.B. (1995). Environmental Chemistry of Soils.

[56] González-Gómez M.A., Belderbos S., Yañez-Vilar S., Piñeiro Y., Cleeren F., Bormans G., Deroose C.M., Gsell W., Himmelreich U., Rivas J. (2019). Development of Superparamagnetic Nanoparticles Coated with Polyacrylic Acid and Aluminum Hydroxide as an Efficient Contrast Agent for Multimodal Imaging. Nanomaterials.

[57] Stevenson F.J. (1994). Humus Chemistry: Genesis, Comoposition, Reactions.

[58] Bieganowski A., Witkowska-Walczak B., Gliński J., Sokołowska Z., Sławinski C., Brzezinska M., Włodarczyk T. (2013). Database of Polish arable mineral soils: A review. Int. Agrophys..

[59] IUSS Working Group WRB (2015). World Reference Base for Soil Resources 2014, Update 2015 International Soil Classification System for Naming Soils and Creating Legends for Soil Maps.

[60] Siek M., Paszko T. (2019). Factors affecting coupled degradation and time-dependent sorption processes of tebuconazole in mineral soil profiles. Sci. Total Environ..

[61] Gregor J.E., Powell H.K.J. (1986). Acid pyrophosphate extraction of soil fulvic-acids. J. Soil Sci..

[62] Wavefunction (2011). Spartan’10 for Windows, Macintosh and Linux. http://downloads.wavefun.com/Spartan10Manual.pdf.

[63] Santos C.B.R., Lobato C.C., Braga F.S., Morais S.S.S., Santos C.F., Fernandes C.P., Brasil D.S.B., Hage-Melim L.I.S., Macêdo W.J.C., Carvalho J.C.T. (2014). Application of Hartree-Fock method for modeling of bioactive molecules using SAR and QSPR. Comput. Mol. Biosci..

[64] Talebpour Z., Ghassempour A., Shamsipur M. (2003). Study of the pesticide naptalam degradation; theoretical and experimental. J. Mol. Struct. THEOCHEM.

[65] Rzeski W., Matysiak J., Kandefer-Szerszeń M. (2007). Anticancer, neuroprotective activities and computational studies of 2-amino-1,3,4-thiadiazole based compound. Bioorgan. Med. Chem..

[66] Singh U.C., Kollman P.A. (1984). An approach to computing electrostatic charges for molecules. J. Comput. Chem..

