# Supplementary material for: Mechanisms of Adsorption of Phenoxyalkanoic Herbicides on Fulvic and Humic Acids
Source: Int J Mol Sci. 2024 Nov 26;25(23):12699. doi: 10.3390/ijms252312699 (PMC11641278; doi:10.3390/ijms252312699)
Supplement: Supplementary file 1 [file ijms-25-12699-s001.zip › ijms-3313382-supplementary.pdf]

Table S1. Mulliken charge of atoms calculated by the Hartree–Fock method at 6-311+G** level.

**PAAH**	**molecule**	**anion**
**2,4-DB**	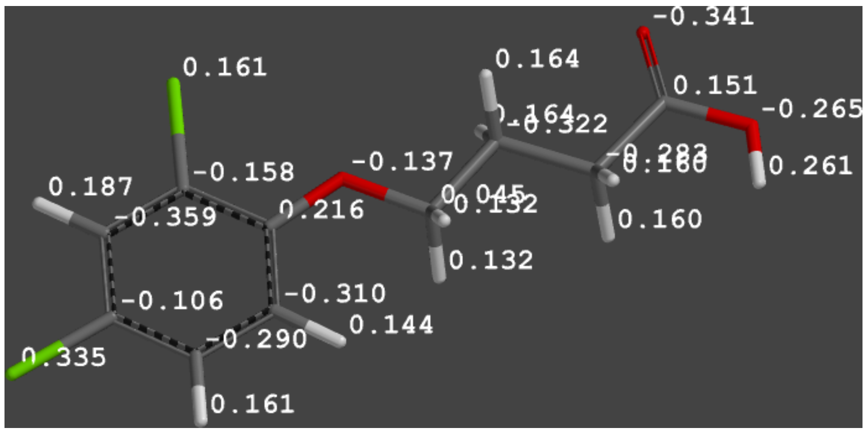	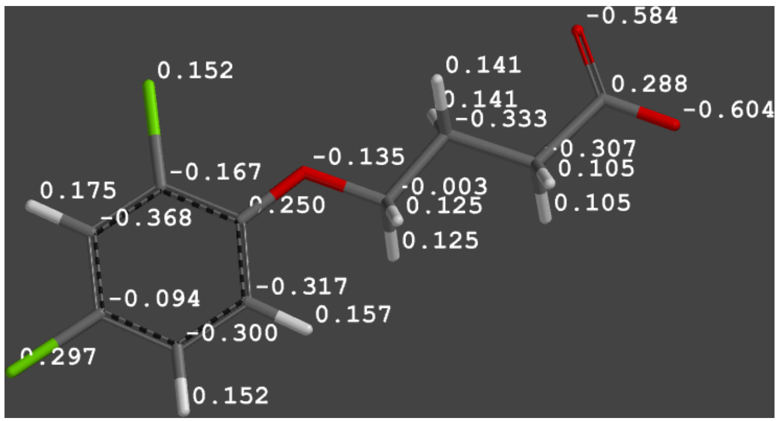

DCPP-P

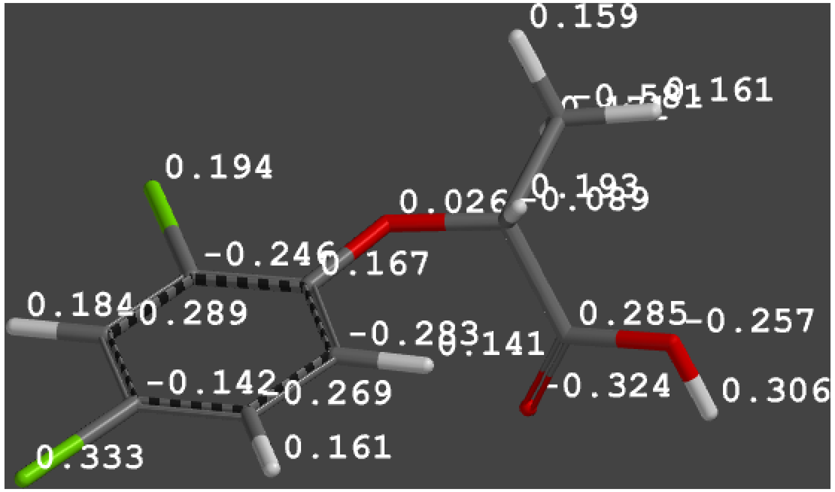
0.159
0.161
0.194
0.026
0.193
-0.089
-0.246
0.167
0.184
0.289
-0.283
0.141
0.285
-0.257
-0.142
0.269
-0.324
0.306
0.333
0.161

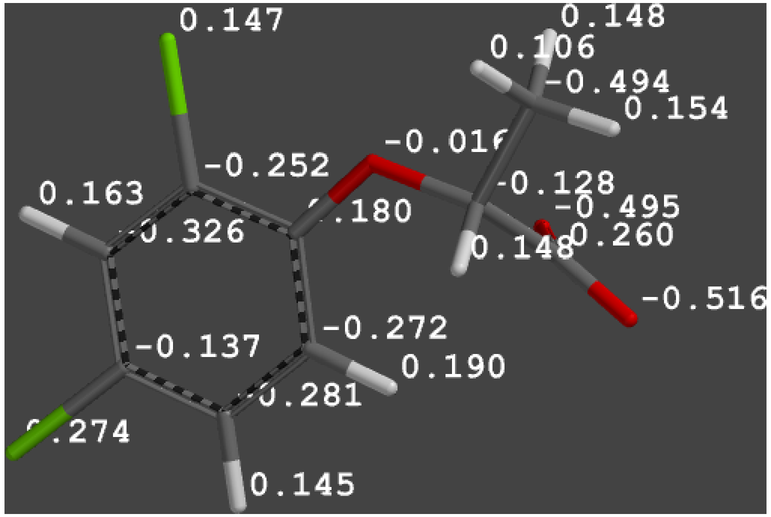
0.147
0.148
0.106
-0.494
0.154
-0.016
-0.252
0.163
.180
-0.128
-0.495
0.260
.326
0.148
-0.516
-0.272
-0.137
0.190
.281
.274
0.145

2,4-D

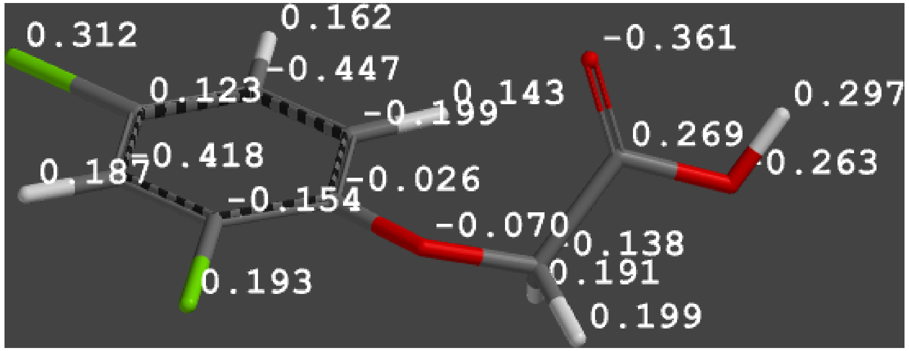
0.312
0.162
-0.361
-0.447
0.123
0.143
0.297
0.199
0.269
0.187
0.418
-0.026
-0.263
-0.154
-0.070
0.138
0.191
0.193
0.199

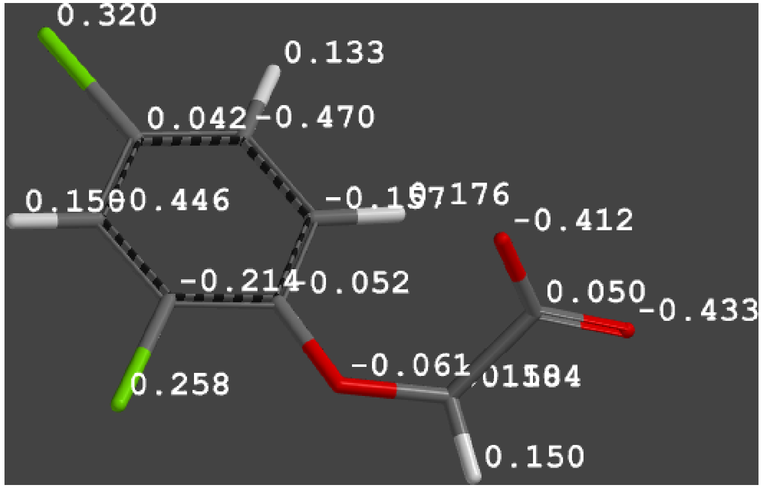
0.320
0.133
0.042
-0.470
0.446
-0.412
-0.214
0.052
0.050
-0.433
-0.061
0.258
0.150

MCPB

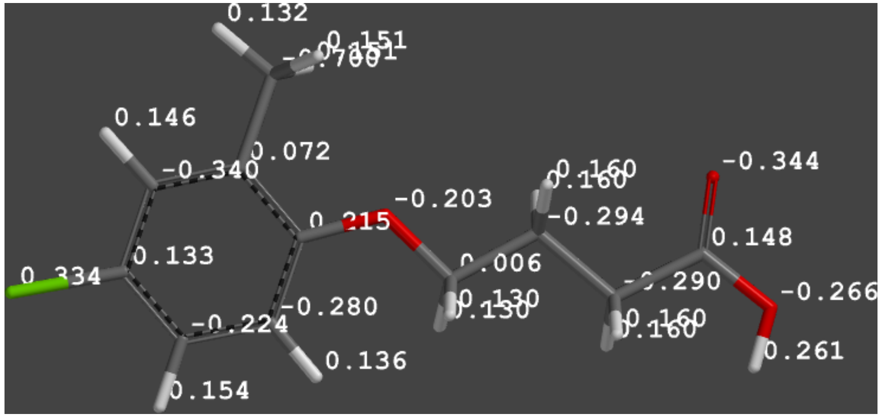

0.132
0.146
-0.340
0.072
-0.203
-0.294
-0.344
0.133
0.148
-0.280
-0.224
-0.266
0.136
0.261
0.154

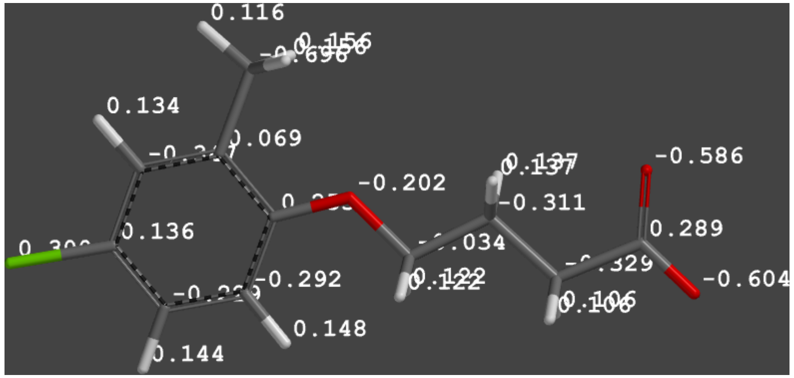

0.116
0.134
0.069
-0.202
-0.311
-0.586
0.136
0.289
-0.292
-0.604
0.148
0.144

MCPP-P

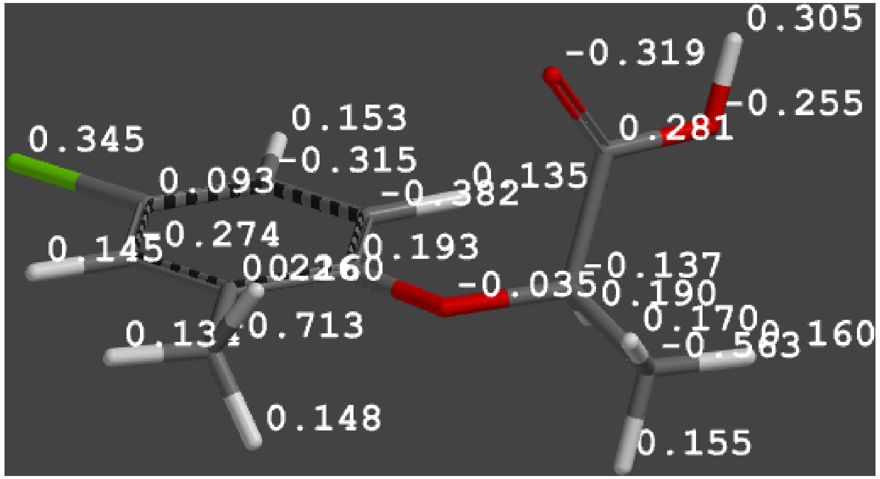

0.305
-0.319
-0.255
0.153
0.345
-0.315
0.281
0.093
0.274
0.193
0.145
-0.137
0.713
0.148
0.155

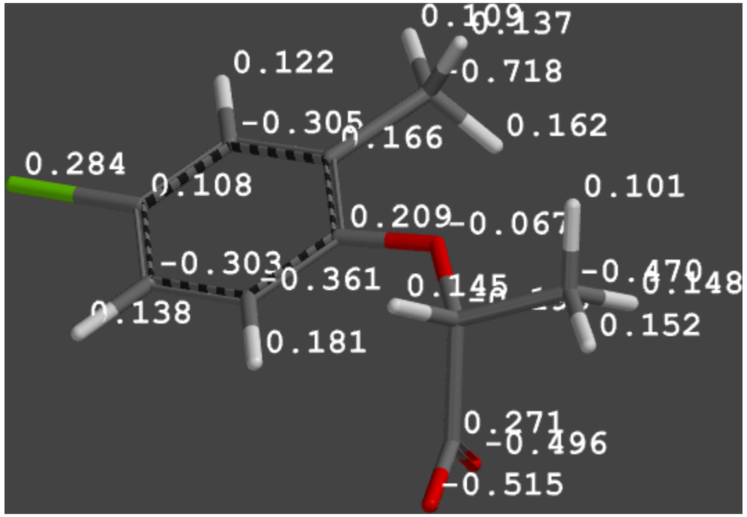

0.122
-0.718
0.162
0.284
0.108
0.101
-0.303
0.361
0.138
0.181
0.152
-0.496
-0.515

MCPA

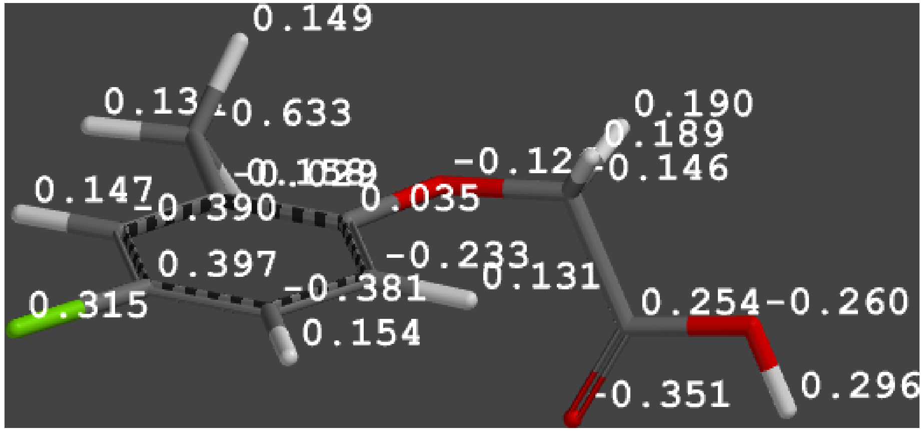
0.149
-0.633
0.190
0.189
-0.146
0.147
-0.390
0.035
0.397
-0.233
-0.381
0.131
0.315
0.154
0.254
-0.260
-0.351
0.296

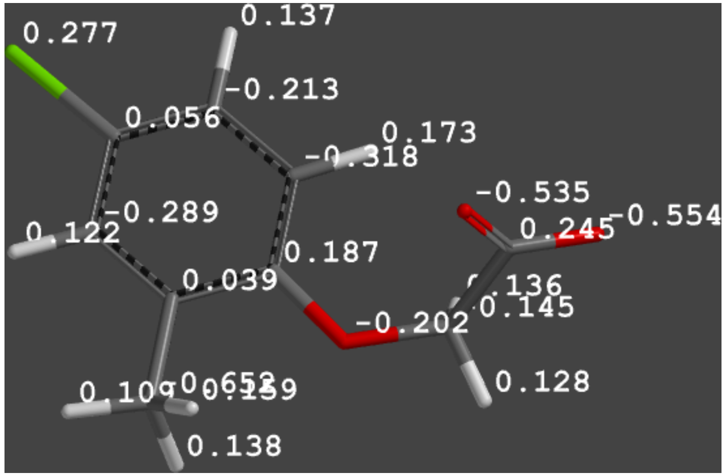
0.277
0.137
-0.213
0.056
0.173
-0.535
-0.289
0.245
-0.554
0.122
0.187
0.039
0.145
-0.202
0.128
0.138
